# A genomic and transcriptomic study toward breast cancer

**DOI:** 10.3389/fgene.2022.989565

**Published:** 2022-10-12

**Authors:** Shan Wang, Pei Shang, Guangyu Yao, Changsheng Ye, Lujia Chen, Xiaolei Hu

**Affiliations:** ^1^ Department of Breast Surgery, Nanfang Hospital, Southern Medical University, Guangzhou, China; ^2^ Department of Critical Care Medicine, Nanfang Hospital, Southern Medical University, Guangzhou, China

**Keywords:** breast cancer, protein–protein interaction, signal pathway, microarray, survival

## Abstract

**Background:** Breast carcinoma is well recognized to be having the highest global occurrence rate among all cancers, being the leading cause of cancer mortality in females. The aim of this study was to elucidate breast cancer at the genomic and transcriptomic levels in different subtypes so that we can develop more personalized treatments and precision medicine to obtain better outcomes.

**Method:** In this study, an expression profiling dataset downloaded from the Gene Expression Omnibus database, GSE45827, was re-analyzed to compare the expression profiles of breast cancer samples in the different subtypes. Using the GEO2R tool, different expression genes were identified. Using the STRING online tool, the protein–protein interaction networks were conducted. Using the Cytoscape software, we found modules, seed genes, and hub genes and performed pathway enrichment analysis. The Kaplan–Meier plotter was used to analyze the overall survival. MicroRNAs and transcription factors targeted different expression genes and were predicted by the Enrichr web server.

**Result:** The analysis of these elements implied that the carcinogenesis and development of triple-negative breast cancer were the most important and complicated in breast carcinoma, occupying the most different expression genes, modules, seed genes, hub genes, and the most complex protein–protein interaction network and signal pathway. In addition, the luminal A subtype might occur in a completely different way from the other three subtypes as the pathways enriched in the luminal A subtype did not overlap with the others. We identified 16 hub genes that were related to good prognosis in triple-negative breast cancer. Moreover, *SRSF1* was negatively correlated with overall survival in the Her2 subtype, while in the luminal A subtype, it showed the opposite relationship. Also, in the luminal B subtype, *CCNB1* and *KIF23* were associated with poor prognosis. Furthermore, new transcription factors and microRNAs were introduced to breast cancer which would shed light upon breast cancer in a new way and provide a novel therapeutic strategy.

**Conclusion:** We preliminarily delved into the potentially comprehensive molecular mechanisms of breast cancer by creating a holistic view at the genomic and transcriptomic levels in different subtypes using computational tools. We also introduced new prognosis-related genes and novel therapeutic strategies and cast new light upon breast cancer.

## Introduction

Breast carcinoma is well recognized to be having the highest global occurrence rate among all types of cancers, being the leading cause of cancer mortality in females worldwide (Ferlay et al., 2021). In the United States, it is estimated that approximately 281550 new female cases were diagnosed in 2021, and it accounted for 15% of estimated deaths due to cancer among women (Siegel et al., 2021). It is well known that breast cancer, which harbors high biological heterogeneity both between and within tumors, is not a single disease and can be classified into four subtypes according to the molecular types, such as luminal A, luminal B, Her2-overexpressed, and triple-negative breast cancer (TNBC) (Perou et al., 2000). Luminal A and luminal B subtypes express the hormone receptors and have a better prognosis than the other two subtypes (Harbeck et al., 2019). The Her2-overexpressed subtype only has Her2 expression and lacks expression of the estrogen receptor (ER) and progesterone receptor (PR), and this subtype has achieved tremendous clinical success because of effective therapy targeting Her2 (Cancer Genome Atlas Network, 2012; Harbeck et al., 2019). TNBC is characterized by the absence of ER, PR, and Her2 expression, which possesses distinct molecular traits and unique recurrence and metastatic patterns (Sørlie et al., 2001; Nielsen et al., 2004; Harbeck et al., 2019).

Currently, the clinical approach to treating breast cancer has been mainly composed of surgery, radiotherapy, chemotherapy, endocrine treatment, and targeted therapy (Harbeck et al., 2019). Although the treatment has been relatively perfect, the reduction of decline in the death rate for breast cancer slowed in females over the past decade, which suggests that we should elucidate the pathogenesis, occurrence, and development of cancer more accurately and find new potential prognostic biomarkers so that we can ensure early diagnosis and develop more personalized treatments and precision medicine to obtain better outcomes (Ferlay et al., 2021). For this to be possible, we think that it is sensible to get a holistic view of the mechanism of breast cancer with system biology approaches. By analyzing high-throughput data extracted from omics data, these approaches present an opportunity to depict the behavior of networks and offer novel therapeutics.

Previous studies have mostly analyzed the molecular mechanisms by comparing the difference between tumor and normal tissues of the breast or focused only on one subtype of breast cancer (Yang et al., 2019; Lin et al., 2020; Liu S. et al., 2020). Actually, during clinical treatment, different measures will be performed according to their subtype, so it is inappropriate to consider different subtypes as a whole to analyze. Also, focusing only on a single subtype cannot help us identify the difference and similarities among different subtypes. In addition, most studies are limited to exploring biomarkers and do not combine the genome with the transcriptome for further exploration.

In this study, we preliminarily delved into the potentially comprehensive molecular mechanisms of breast cancer by creating a holistic view at the genomic and transcriptomic level in four different subtypes using computational tools. To the best of our knowledge, this is the first time such a systematic biological study was performed on breast cancer according to its subtypes and at the genomic and transcriptomic level. We also first explored genes such as *SRSF1*, *BUB1B*, *KIF23*, *HNRNPF*, and *ELAVL1* and obtained an exact result in our study. We re-analyzed the dataset deposited by Gruosso et al, (2016) and exhibited considerable protein–protein interaction networks. In addition, we performed network, clustering, and functional analysis so that we could have a deep understanding of the central genes of each subtype. Otherwise, pathways of different subtypes were identified with enrichment analysis, and new micro-RNAs (miRNAs) and transcription factors (TFs) were introduced to assay the regulatory mechanisms of differential expression genes (DEGs).

## Materials and methods

### Microarray data and DEG screening

A microarray dataset with accession number “GSE45827” from the GEO database was downloaded (Gruosso et al., 2016). This dataset includes 14 cell line samples, 41 TNBC cancer samples, 30 Her2 cancer samples, 29 luminal A cancer samples, 30 luminal B cancer samples, and 11 normal breast tissue samples, and we only used the cancer samples and normal breast tissue samples to analyze. GEO2R (RRID:SCR_016569, http://www.ncbi.nlm.nih.gov/geo/geo2r/) is an online tool that can be used to screen DEGs across different groups. Using GEO2R of GEO, groups (TNBC vs. normal, Her2 vs. normal, luminal A vs. normal, and luminal B vs. normal) were compared to identify DEGs of the four subtypes. Benjamini–Hochberg false discovery was used for *p*-value adjustment. Genes were declared as DEGs when |lgFC|≥3 and the adjusted *p*-value (adj.p) < 0.01. The heat map was performed by SangerBox online tool version 3.0 (http://www.sangerbox.com/tool), and the volcano plot was drawn by GraphPad Prism for Windows (v9.2.0, RRID:SCR_002798, GraphPad Software, San Diego, California United States, www.graphpad.com).

### PPI network construction

The PPI networks of DEGs were built with the STRING online tool (v11.0, RRID:SCR_005223, https://string-db.org/) (Szklarczyk et al., 2019). DEGs were mapped to the STRING database to estimate the interactive relationships, setting the confidence cutoff to 0.95. Then, Cytoscape (v3.8.2, RRID:SCR_003032) software was used to visualize the PPI network. For network analysis, the MCODE plugin (v2.0.0, RRID:SCR_015828) of Cytoscape software was used to investigate modules, highly connected sub-networks, and seed genes based on default settings (Bader and Hogue, 2003; Shannon et al., 2003). CytoHubba plugin version 0.1 of Cytoscape (v3.8.2, RRID:SCR_003032) was applied to detect hub genes (Chin et al., 2014). The criteria of hub genes were as follows: MCC cutoff =1000, degree cutoff = 10, closeness cutoff = 50, and betweenness cutoff = 1000. In addition, Venn diagrams were drawn by FunRich software (v3.1.3, RRID:SCR_014467).

### Pathway enrichment analysis

Genes clustered with MCODE were analyzed by the Cytoscape ClueGO plugin (v2.5.8, RRID:SCR_005748), choosing Reactome and KEGG databases to retrieve pathways (Kanehisa and Goto, 2000; Bindea et al., 2009; Jassal et al., 2020). Bonferroni step down was used to adjust the *p*-value, and signal pathways with adj.*p* ≤ 0.05 were recognized.

### Survival analysis

The Kaplan–Meier plotter mRNA breast cancer database (RRID:SCR_018753, https://kmplot.com/analysis/), an online database, was used to analyze the overall survival (OS) with hazard ratios (HRs), 95% confidence intervals (95% CIs), and logrank *p*-value. The JetSet best probe set was selected as gene probes. During the prognosis analysis, patients were split into two groups in accord with the auto-select best cutoff. The logrank *p*-value <0.05 was considered to show a statistical significance. The forest plot was drawn using Xiantao scholar (https://www.xiantao.love/), another online platform for data analysis.

### Expression analysis of prognosis-related hub genes

The differential expression of prognosis-related hub genes was analyzed in the GSE45827 dataset and validated in TCGA dataset using the ggplot2 package of R software. During the analysis, the Mann–Whitney U test, Welch’s t-test, and Student’s t-test were used, respectively, depending on the normality and homogeneity of variance. Similarly the *p*-value < 0.05 was considered to show a statistical significance.

### Functional exploration of each prognosis-related hub gene

GeneMANIA (http://www.genemania.org) was used to evaluate the functions of prognosis-related hub genes according to several bioinformatics methods, such as co-expression, physical interaction, prediction, co-localization, and shared protein domains and pathways (Warde-Farley et al., 2010).

### miRNA and TF enrichment analysis

The microRNAs and TFs were predicted using the Enrichr online server (RRID:SCR_001575) (Kuleshov et al., 2016). MiRNAs were predicted by the TargetScan microRNA 2017 database, while TFs were predicted by the ChEA2016 database. Adj.*p* ≤ 0.01 was considered to show statistical significance. The miRNAs with higher combined scores were selected.

## Results

### By analyzing the GEO database, DEGs were identified

**FIGURE 1:**
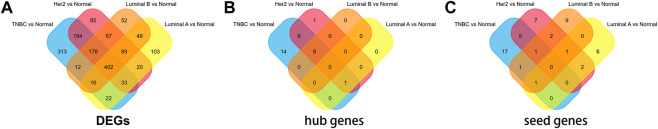
Overlapping of **(A)**DEGs, **(B)**hub genes, and **(C)**seed genes.

**FIGURE 2:**
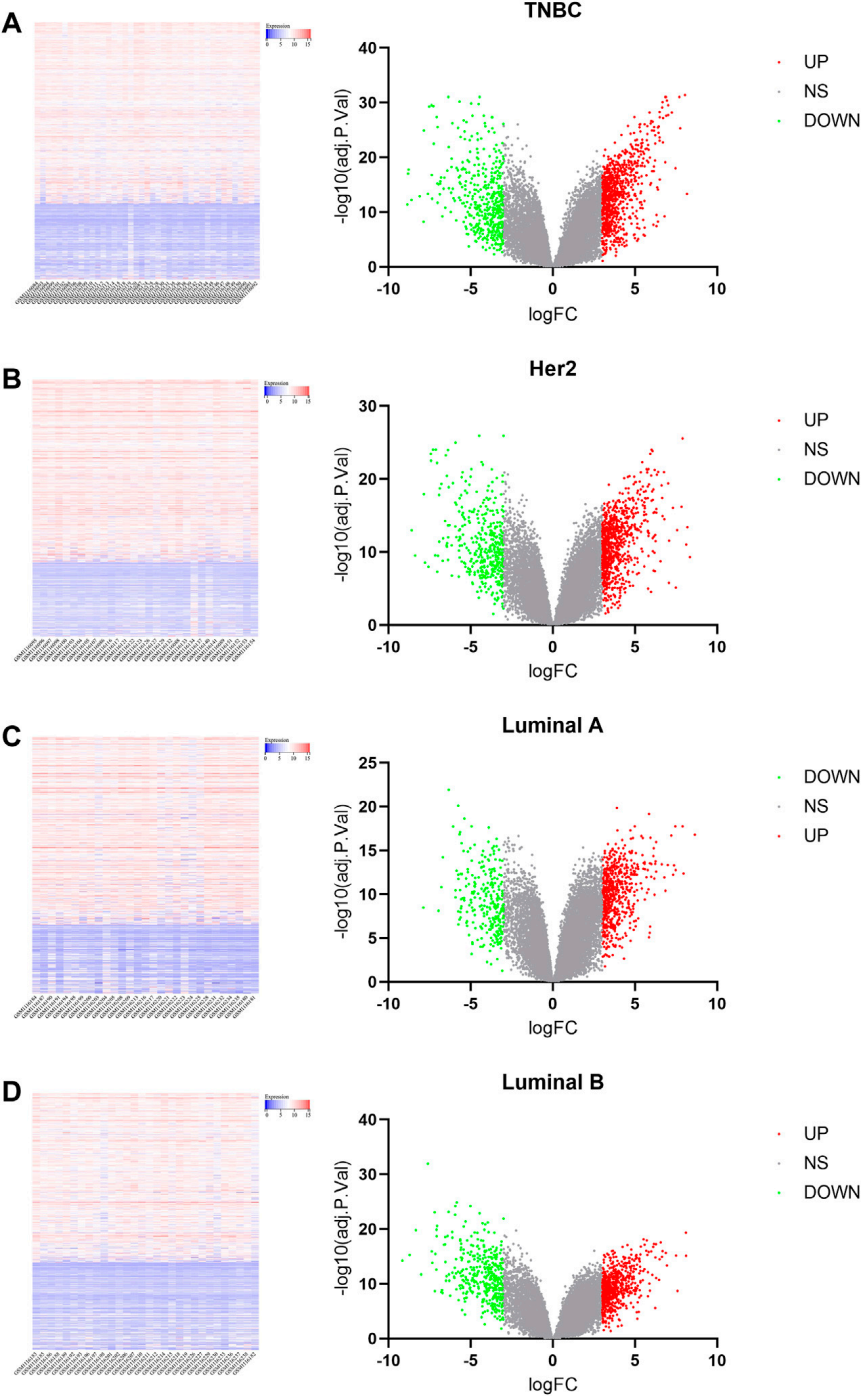
Heat map and volcano plot analysis of DEGs. In the volcano plot, blue dots on the left indicate the downregulated genes, gray dots in the middle indicate genes that are not differentially expressed, and red dots in the right indicate the upregulated genes. [**(A)**: TNBC vs. normal, **(B)** Her2 vs. normal, **(C)** luminal A vs. normal, and **(D)** luminal B vs. normal.]

The microarray dataset “GSE45827” which includes primary invasive breast carcinoma (41 TNBC, 30 Her2, 29 luminal A, and 30 luminal B) and 11 normal tissues has been analyzed. Using the GEO2R tool, we found 1170, 1058, 733, and 854 DEGs which are significantly variably expressed between TNBC vs. normal, Her2 vs normal, luminal A vs. normal, and luminal B vs. normal, respectively. Most of the DEGs overlapped between the four molecular subtypes (Figure 1A). The heat map and volcano plot are shown in Figure 2.

### Protein–protein interaction networks were constructed

**FIGURE 3:**
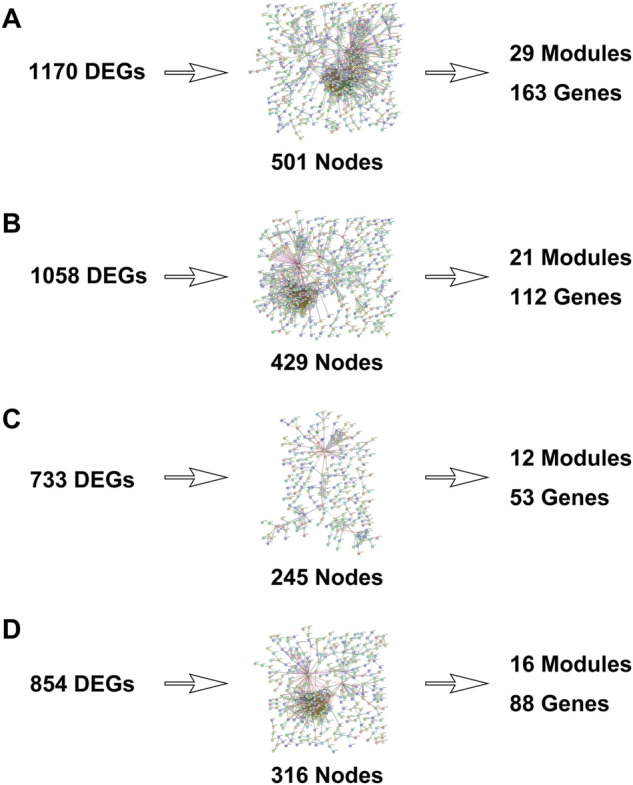
Protein–protein interaction networks were built with differentially expressed genes. [**(A)**: TNBC vs. normal, **(B)** Her2 vs. normal, **(C)** luminal A vs. normal, and **(D)** luminal B vs. normal.]

The PPI networks with DEGs were conducted using the STRING online tool. The edges indicate both functional and physical protein associations. The TNBC subtype has the most nodes and edges. A total of 501, 429, 245, and 316 nodes (genes) are in PPI networks TNBC vs. normal, Her2 vs. normal, luminal A vs. normal, and luminal B vs. normal, respectively (Figures 3A–D). The topological clusters also called modules found in *MCODE* identified groups of genes with a similar function, and each module has the most effective genes, called seed genes. Similarly, the TNBC subtype has the most modules and seed genes. Interestingly, these sets of seed genes in different subtypes exhibited few overlaps (Figure 1C). Network topology was measured based on the graph theory concepts such as MCC, degree, closeness, and betweenness. Seed genes such as *CDC6* and *RFC3* were hub genes in the TNBC. In the Her2 subtype, *AURKB* was identified as both a seed gene and a hub gene. Only *SRSF1* which coincided with the TNBC and Her2 subtype was introduced as a hub gene in the luminal A subtype. All the hub genes in the luminal B subtype overlapped with the TNBC and Her2 subtype (Figure 1B). The hub genes are represented in Table 1.

**TABLE 1:** Hub genes in the PPI network.

ID	MCC	Degree	Closeness	Betweenness	ID	MCC	Degree	Closeness	Betweenness
TNBC vs. normal					Her2 vs. normal				
*CDK1*	9.22E+13	89	192.19008	22078.67341	*NDC80*	9.22E+13	40	140.9357	1013.13909
*CDC5L*	8106206	77	192.6067	69392.04571	*KIF2C*	9.22E+13	34	139.8357	1240.99518
*CCNB1*	9.22E+13	64	178.27341	7504.97904	*KIF11*	9.22E+13	43	145.0857	2836.58396
*CDC20*	9.22E+13	62	165.25675	3752.95365	*CDK1*	9.22E+13	73	163.2691	20555.50241
*BUB1*	9.22E+13	57	172.85675	3211.85824	*CDC20*	9.22E+13	52	138.8952	2567.19563
*PLK1*	9.22E+13	57	171.69008	4346.90413	*CCNB2*	9.22E+13	47	136.4191	1209.68801
*CCNB2*	9.22E+13	56	160.54008	2061.86361	*CCNB1*	9.22E+13	50	149.0857	3631.78964
*CCNA2*	9.22E+13	55	161.05675	4757.15522	*CCNA2*	9.22E+13	44	134.2286	2002.89489
*BUB1B*	9.22E+13	52	168.52341	5703.00881	*BUB1B*	9.22E+13	45	145.3357	4540.65528
*AURKB*	9.22E+13	51	168.02341	2567.01982	*BUB1*	9.22E+13	49	148.6691	3185.181
*KIF11*	9.22E+13	48	167.77341	2555.27954	*AURKB* *^a^*	9.22E+13	46	145.8357	3137.25928
*NDC80*	9.22E+13	47	165.22341	1237.29857	*KIF23*	5.50E+12	27	131.3333	9398.7775
*KIF2C*	9.22E+13	42	163.02341	1465.60477	*UBE2C*	1.07E+12	31	123.4036	2928.73296
*NUF2*	9.22E+13	35	155.40317	1007.52523	*CDC5L*	822688	65	163.5071	53316.02571
*ASPM*	9.22E+13	34	145.35913	1083.30296	*RACGAP1*	8.86E+07	22	141.2333	18065.07384
*KIF23*	9.22E+13	31	149.33095	11155.80535	*ECT2*	41048	11	123	4077.48928
*CDC6* *^a^*	47892	27	140.84127	1232.15458	*SRSF1*	1082	12	112.3119	2071.32644
*MCM2*	8932	26	141.43889	3024.46868	Luminal A vs. normal				
*MCM4*	9062	25	141.10556	1725.51468	*SRSF1*	1034	11	51.00238	1044.8
*PRC1*	3.82E+11	24	141.62341	1039.07441	Luminal B vs. normal				
*RACGAP1*	1.25E+10	24	159.54762	17261.72834	*CDK1*	1.57E+11	57	110.2952	6869.988
*PCNA*	1845	23	139.58889	2598.0432	*CDC20*	1.57E+11	45	96.84524	1313.656
*CHEK1*	6622	20	148.45317	8464.48184	*BUB1*	1.57E+11	42	102.3786	1944.034
*RPA1*	2406	18	138.29603	2279.10647	*BUB1B*	1.27E+11	38	99.2119	1242.749
*RFC3* *^a^*	1648	15	146.13651	1025.54515	*CCNB1*	7.79E+10	40	100.7119	1303.47
*SRSF1*	1826	15	131.52937	1327.95608	*RACGAP1*	987987	19	94.56667	4407.43
*FOXM1*	6673	13	132.69881	3099.92655	*KIF11*	1.56E+11	37	98.87857	1629.572
*ECT2*	367926	11	138.58095	4860.54991	*KIF23*	2.62E+09	23	90.05	3930.105
*HNRNPF*	1729	11	128.79603	2045.45414	*CDC5L*	26009	47	109.5952	21827.75
*ELAVL1*	1693	11	128.62937	1102.88098					

a Seed genes.

### Pathway enrichment analysis was performed

**FIGURE 4:**
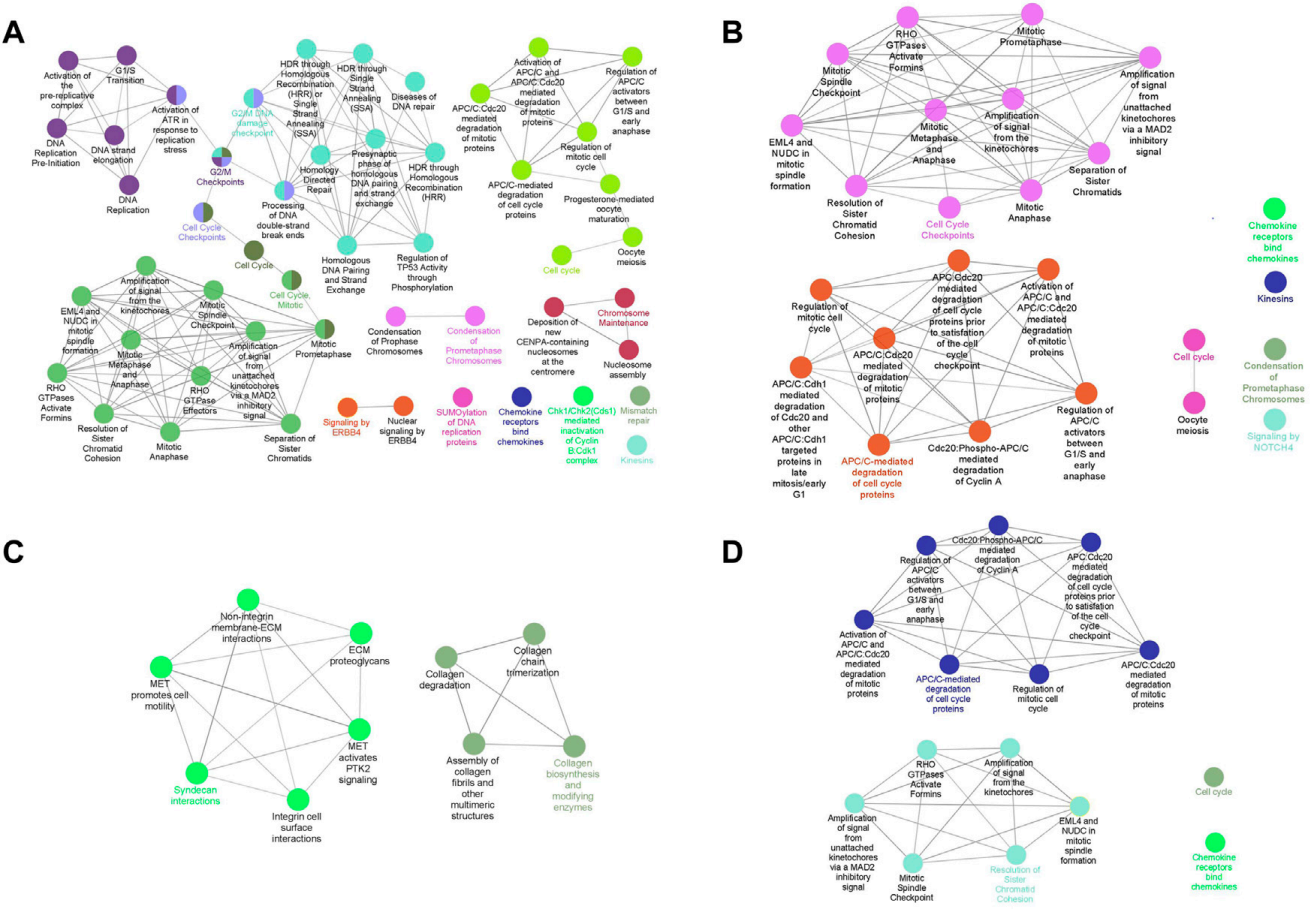
Pathway enrichment analysis of clustered genes. Interconnected and informative pathways mainly are indicated by identical colors. The most significant pathway in each network is labeled. [**(A)**: TNBC vs. normal, **(B)** Her2 vs. normal, **(C)** luminal A vs. normal, and **(D)** luminal B vs. normal].

The pathway enrichment analysis was executed based on genes identified by MCODE. We reached 51, 25, 10, and 15 pathways by performing the pathway enrichment analysis from 163, 112, 53, and 88 genes, respectively (Figure 4). The genes used to analyze pathways were those that were included in PPI modules. The pathways involved in TNBC were mainly about DNA replication, DNA repair, and mitosis, while in the Her2 and luminal B types, they were mitosis, and most of the pathways involved in Her2 and luminal B subtypes were included in the TNBC subtype. In addition, the pathways that play a role in luminal A were totally different from the other three subtypes, especially associated with extracellular matrix organization and collagen formation. In the TNBC subtype, the top three pathways that contain the most genes are a condensation of prometaphase chromosomes, Chk1/Chk2(Cds1)-mediated inactivation of cyclin, and activation of ATR in response to replication stress. In the Her2 subtype, condensation of prometaphase chromosomes, resolution of sister chromatid cohesion, and amplification of signals from the kinetochores are the top three pathways. Syndecan interactions, MET-activated PTK2 signaling, and MET-promoted cell motility are the top three pathways in the luminal A subtype. In the luminal B subtype, the top three pathways are an amplification of the signal from the kinetochores, amplification of the signal from unattached kinetochores *via* a MAD2 inhibitory signal, and resolution of sister chromatid cohesion. Furthermore, there were also some pathways that are unique to specific subtypes, for example, the ERBB4 pathway in TNBC and the NOTCH4 pathway in luminal B.

### Survival analysis of hub genes in different subtypes was carried out

**FIGURE 5:**
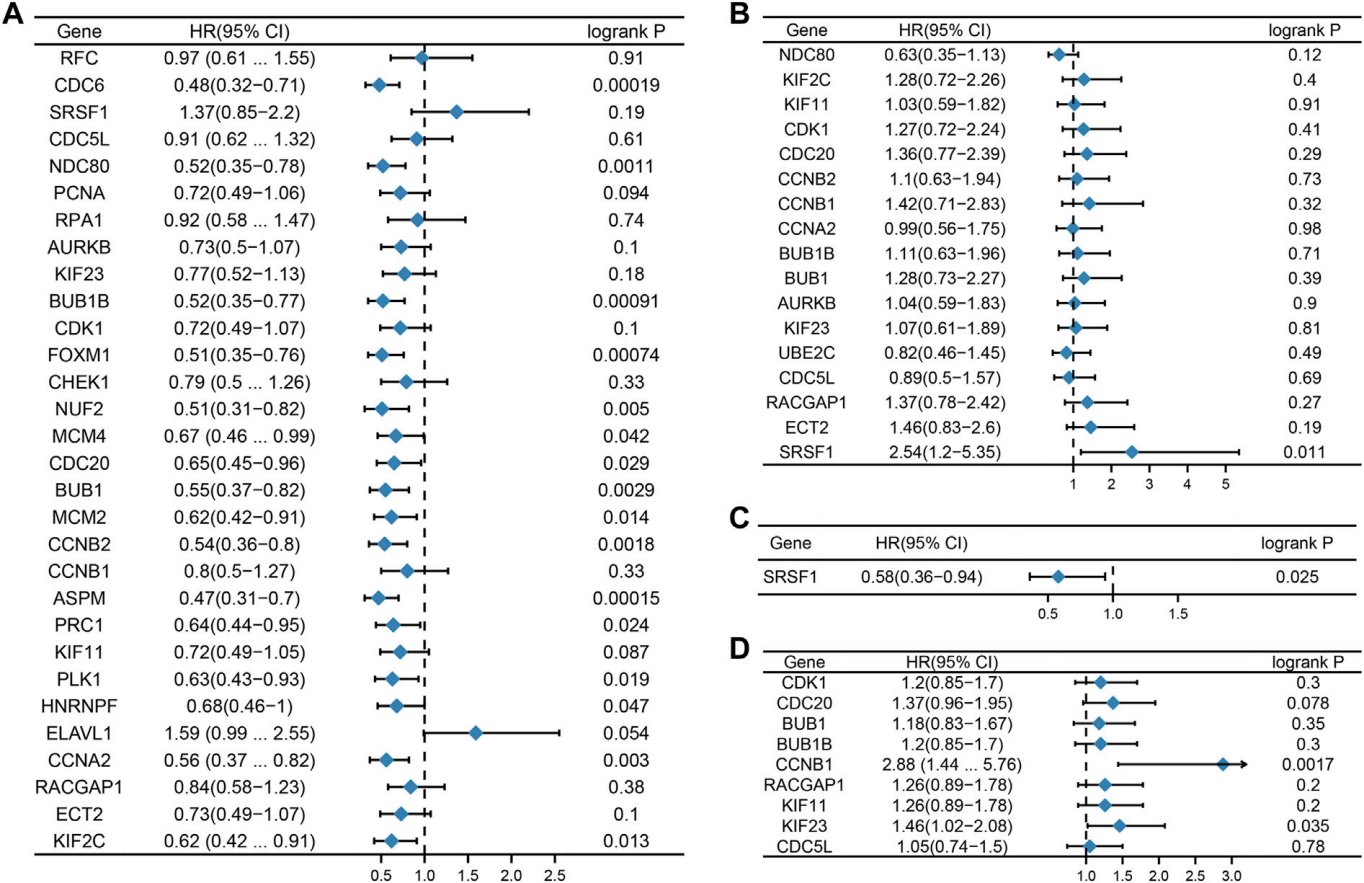
Prognostic value of hub genes. Forest plots show the correlation between hub gene expression and prognosis in different subtypes of breast cancer. [**(A)**: TNBC, **(B)** Her2, **(C)** luminal A, and **(D)** luminal **(B)**.]

We then considered whether the hub genes in the different subtypes of breast cancer were associated with prognosis. The relationship between hub gene expression and survival rates was evaluated using the Kaplan Meier plotter. The prognostic analysis demonstrated that hub genes such as *CDC6*, *NDC80*, *BUB1B*, *FOXM1*, *NUF2*, *MCM4*, *CDC20*, *BUB1*, *MCM2*, *CCNB2*, *ASPM*, *PRC1*, *PLK1*, *HNRNPF*, *CCNA2*, and *KIF2C* were related to good prognosis in TNBC (Figure 5A). In addition, *SRSF1* was negatively correlated with overall survival (OS) in the Her2 subtype, while in the luminal A subtype, it showed the opposite relationship (Figures 5B,C). In the luminal B subtype, CCNB1 and KIF23 were associated with poor prognosis (Figure 5D).

### The expression of prognosis-related hub genes in each subtype was analyzed in the GSE45827 dataset and validated in another independent dataset

**FIGURE 6:**
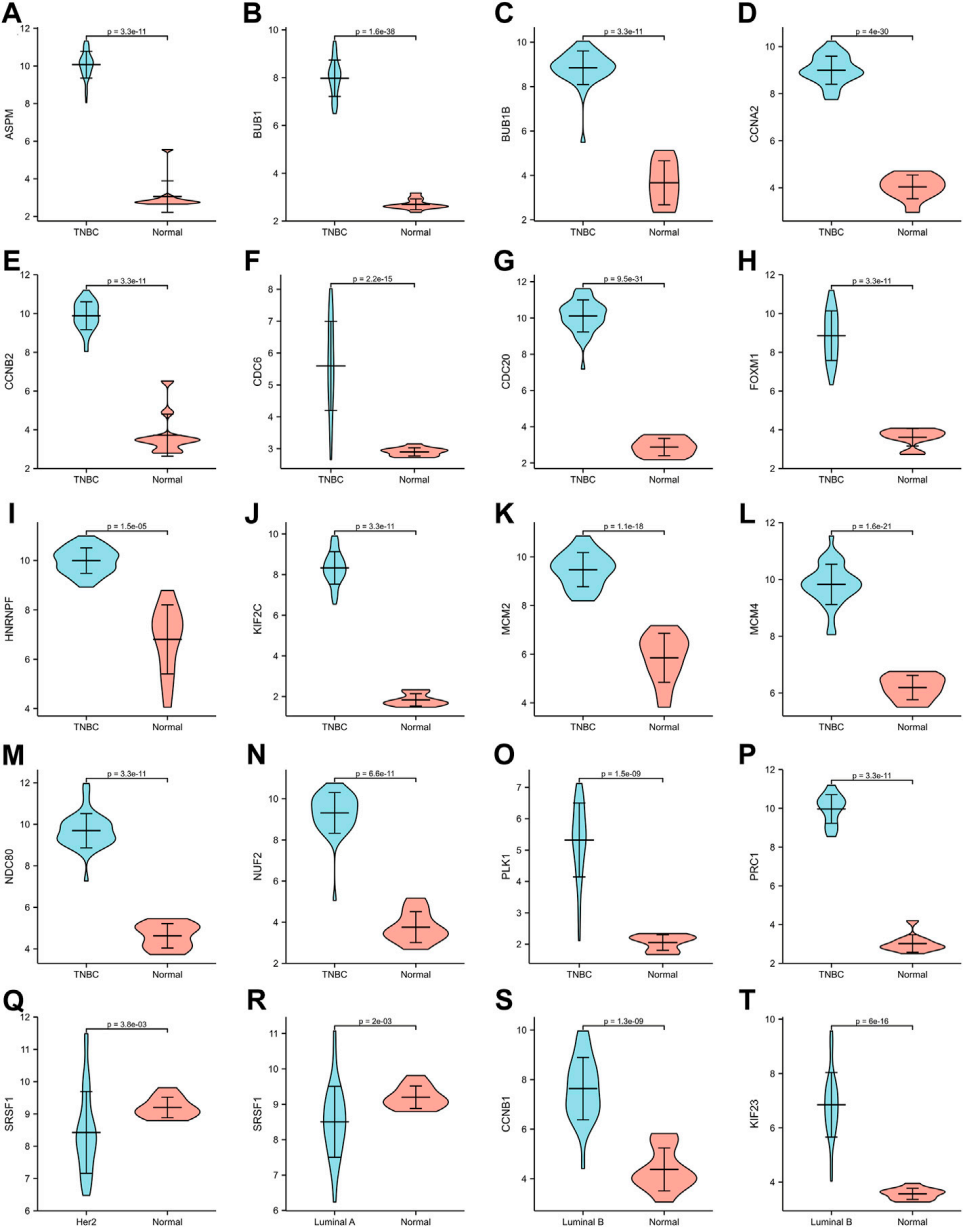
Expression of hub genes in GSE45827. **(A–P)** *ASPM*, *BUB1*, *BUB1B*, *CCNA2*, *CCNB2*, *CDC6*, *CDC20*, *FOXM1*, *HNRNPF*, *KIF2C*, *MCM2*, *MCM4*, *NDC80*, *NUF2*, *PLK1*, *PRC1* expression in TNBC subtype. **(Q)** *SRSF1* expression in Her2 subtype. **(R)** *SRSF1* expression in Luminal A subtype. **(S,T)** CCNB1, KIF 23 expression in Luminal B subtype.

**FIGURE 7:**
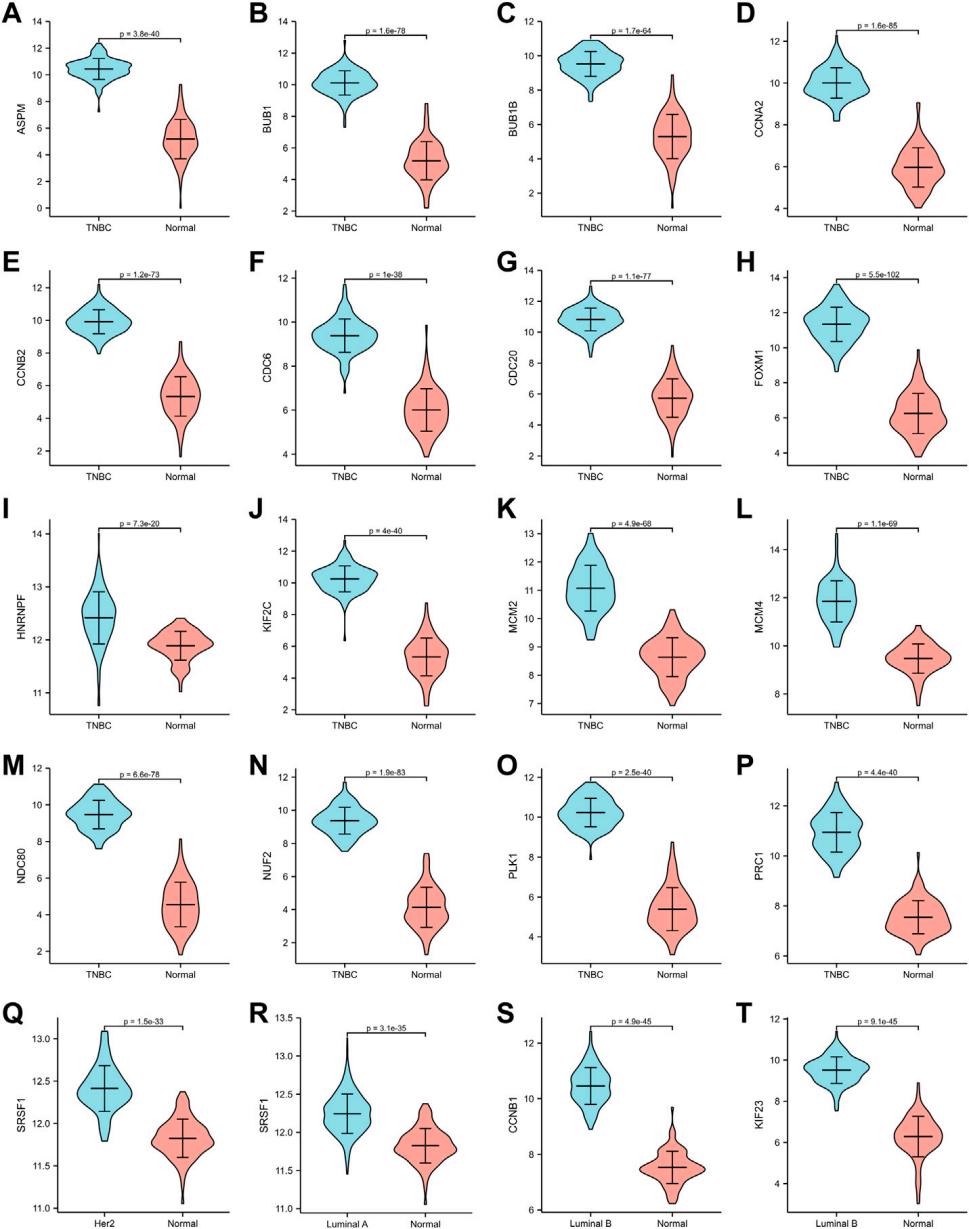
Validation of hub genes in TCGA dataset. **(A–P)** *ASPM*, *BUB1*, *BUB1B*, *CCNA2*, *CCNB2*, *CDC6*, *CDC20*, *FOXM1*, *HNRNPF*, *KIF2C*, *MCM2*, *MCM4*, *NDC80*, *NUF2*, *PLK1*, *PRC1* expression in TNBC subtype. **(Q)** *SRSF1* expression in Her2 subtype. **(R)** *SRSF1* expression in Luminal A subtype. **(S,T)** CCNB1, KIF 23 expression in Luminal B subtype.

The expression of hub genes that were related to prognosis in GSE45827 was analyzed using ggplot2 of R software. All the hub genes that were analyzed in TNBC were upregulated, including *ASPM*, *BUB1*, *BUB1B*, *CCNA2*, *CCNB2*, *CDC6*, *CDC20*, *FOXM1*, *HNRNPF*, *KIF2C*, *MCM2*, *MCM4*, *NDC80*, *NUF2*, *PLK1*, and *PRC1* (Figure 6A–P). *SRSF1* was a hub gene that was related to prognosis in both Her2 and Luminal A subtypes, and its expression profiles in the two subtypes were similar—it had a higher expression level in the normal tissues than in the tumor tissue (Figure6Q–6R). The two hub genes that were associated with prognosis in the luminal B subtype were also upregulated (Figure 6S–6T). Then, we validated the expression of prognosis-related hub genes in TCGA dataset. The expression of SRSF1 in Her2 and luminal A subtypes is not consistent with the result of the GEO profile analysis, and it was highly expressed in tumor tissues rather than normal tissues (Figure 7Q–7R). The expression levels of the rest hub genes were in accordance with the GEO profile analysis (Figure 7A–P, Figure 7S,T).

### Potential functions for each prognosis-related hub gene were explored

**FIGURE 8:**
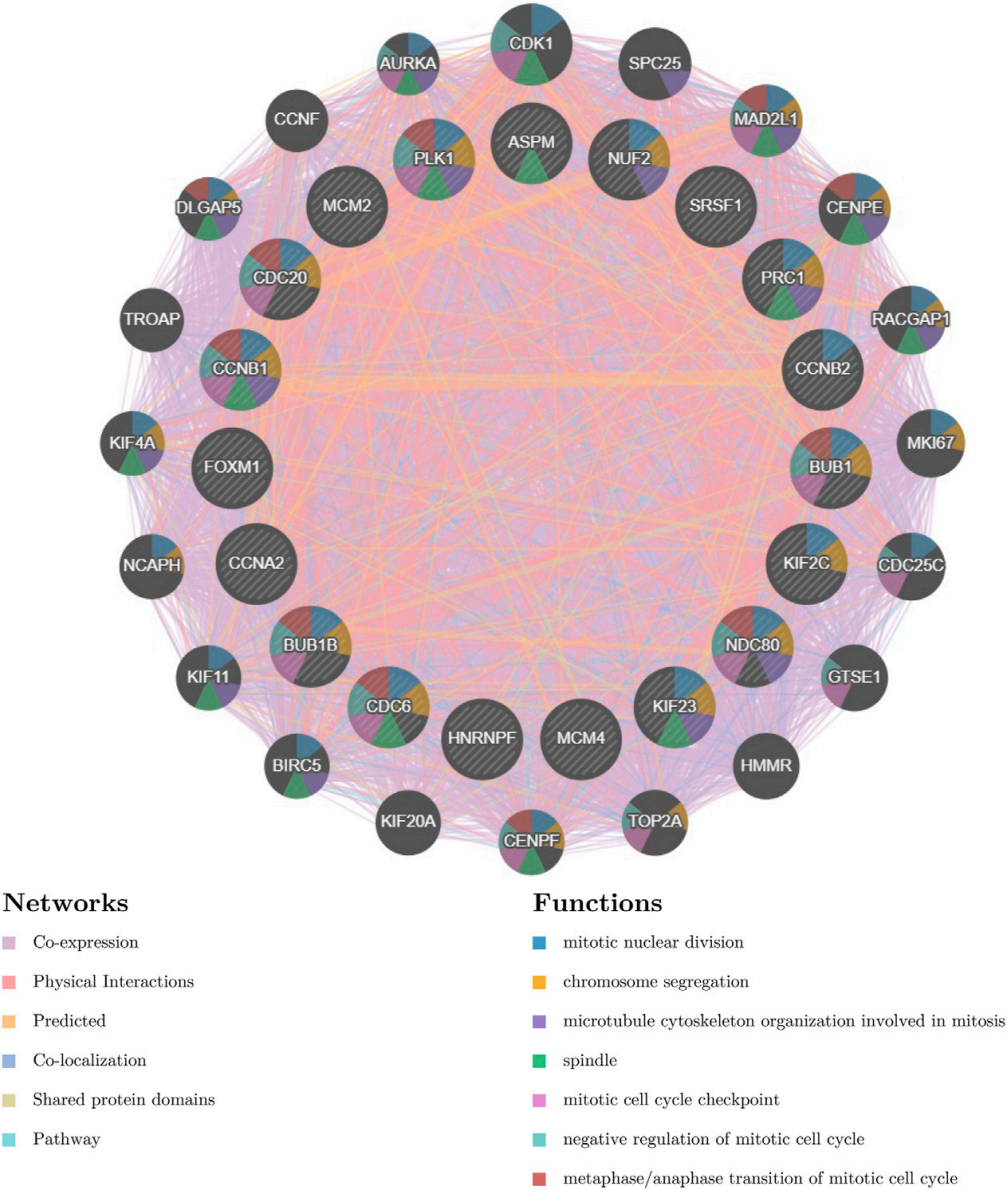
Protein–protein interaction network (GeneMANIA) of prognosis-related hub genes.

We then investigated the functions of the prognosis-related hub genes using GeneMANIA. It showed that these genes were correlated with mitotic nuclear division (FDR=2.13e-33), chromosome segregation (FDR=1.68e-24), microtubule cytoskeleton organization involved in mitosis (FDR=8.79e-20), spindle (FDR=8.94e-18), mitotic cell cycle checkpoint (FDR=9.00e-17), negative regulation of the mitotic cell cycle (FDR=3.42e-14), and metaphase/anaphase transition of the mitotic cell cycle (FDR=3.00e-17) (Figure 8).

### miRNAs and TFs enriched with DEGs were determined

**FIGURE 9:**
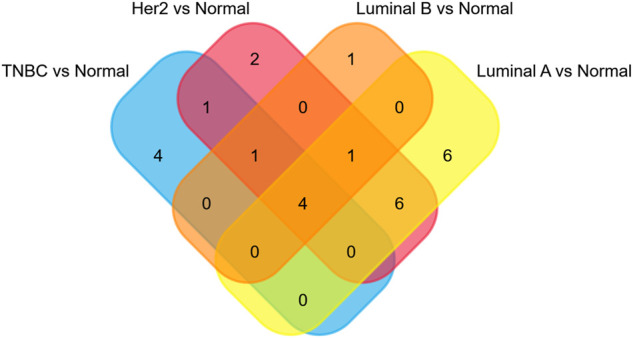
Overlapping of TFs.

**FIGURE 10:**
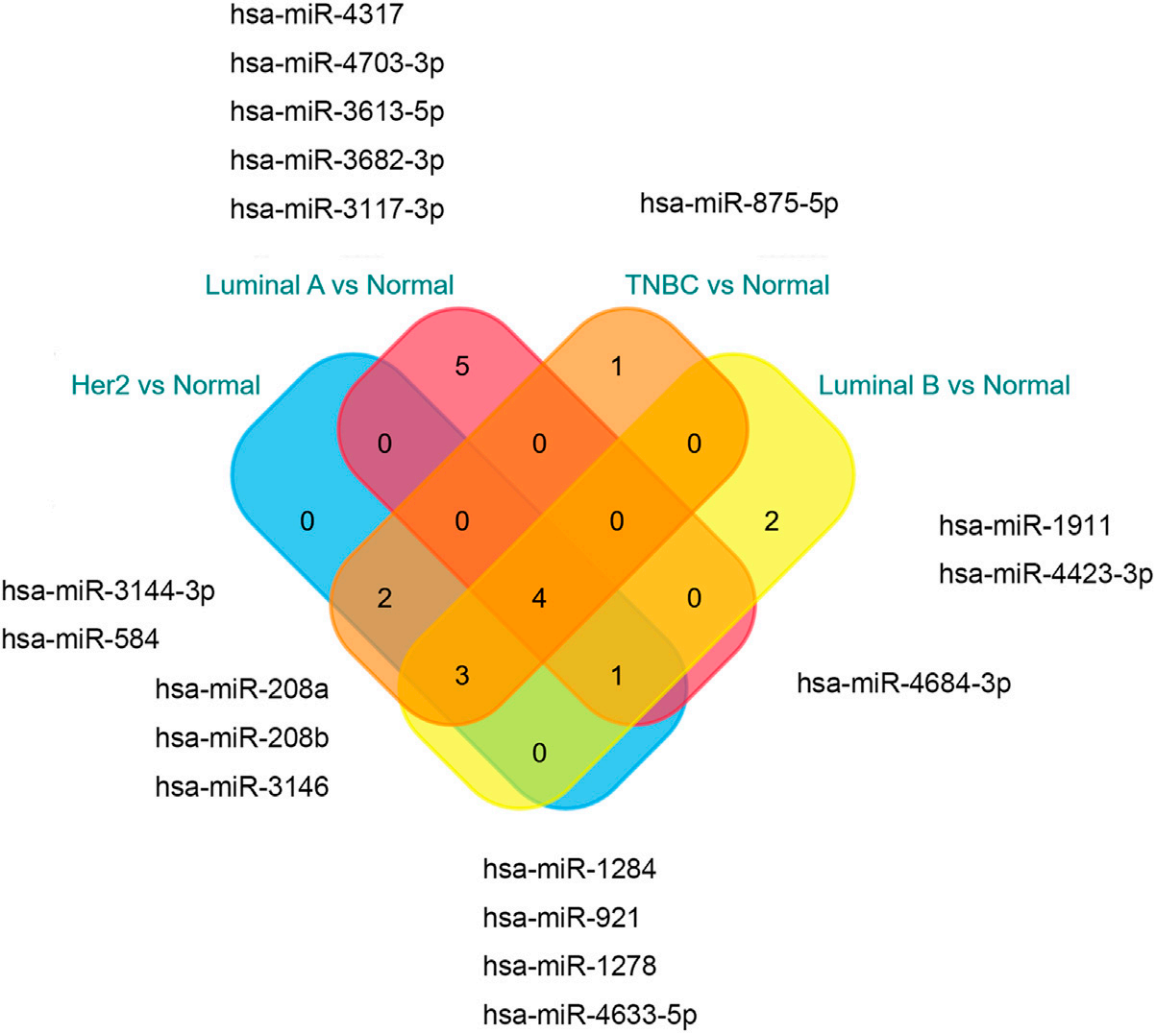
miRNA enrichment analysis results.

The miRNAs and TFs, as important regulators of DEGs, were predicted using the Enrichr web server. It is worth noting that the luminal A subtype had the most TFs, while it had the least hub genes among the four molecular subtypes. Seven TFs exerted their function in all four subtypes (Figure 9). TFs that were meaningful in breast cancer development are shown in Table 2. The top 10 miRNAs enriched with DEGs in each subtype are also shown (Figure 10).

**TABLE 2:** Transcription factor enrichment analysis.

TNBC vs. normal	Her2 vs. normal	Luminal A vs. normal	Luminal B vs. normal
E2F4	CLOCK	CLOCK	CLOCK
AR	FOXM1	FOXM1	FOXM1
CLOCK	RELA	ZNF217	RELA
RELA	POU3F2	SOX2	BACH1
E2F7	BACH1	WT1	SOX2
BACH1	ZNF217	RELA	EOMES
CHD1	AR	CJUN	AR
KDM6A	SOX2	AHR	
E2F1	FOXA1	ESR1	
FOXM1	PIAS1	AR	
	E2F7	ESR2	
	AHR	ARNT	
	CJUN	NR3C1	
	WT1	PIAS1	
	SMAD4	RUNX1	
		PPAR	
		SMAD4	

## Discussion

In the present study, bioinformatic approaches were carried out to show the DEGs, modules, seed genes, PPI, and hub genes in each subtype (TNBC, Her2, luminal A, and luminal B). The topological clusters which have high-density regions in the network, also called modules, find in MCODE-identified groups of genes with a similar function. Genes in the highly interconnected subnetwork modules are expected to be involved in the same pathways or in roles with related biological functions. Each module has a most effective gene which has high centrality, named seed genes. The nodes in the PPI network represent genes, while the edges indicate both functional and physical protein associations. Nodes with high degree, betweenness, closeness, and MCC are significant for the network and are called hub genes which can serve as targets. The analysis of these elements implied that the carcinogenesis and development of TNBC were the most important and complicated processes in breast carcinoma, occupying the most DEGs, modules, seed genes, hub genes, and the most complex PPI network.

The role of some of those hub genes that we identified in our study has been verified in breast cancer, such as UBE2C whose overexpression plays a critical role in the incidence and development of breast cancer, and such a therapeutic strategy that combines palbociclib with tamoxifen might be promising in patients with HR+/HER2-breast cancer overexpressing UBE2C (Mo et al., 2017; Kim et al., 2019). Otherwise, it is reported that MCM2 and MCM4, which have a higher expression in high histological grade breast cancer, may be used as useful parameters to distinguish luminal A and luminal B subtypes instead of ki-67 and are related to poor prognosis (Issac et al., 2019), which is partly consistent with our results in the TNBC subtype. Also, high expression of *RACGAP1* is supposed to be not only a strong poor prognostic marker in luminal-like breast cancer but might also be a predictor of response to treatment with tamoxifen and adjuvant chemotherapy (Milde-Langosch et al., 2013). In addition, the expression levels of *CDK1* and *CCNA2* have been previously revealed to be considerably higher in breast cancer tissues than those in normal tissues, and these genes lead to breast cancer development and are related to poor prognosis (Xing et al., 2021); however, compared with their study, we found that CDK1 had an independent association with prognosis, and the discrepancy may be due to different analysis methods, as we analyzed according to the subtype while they did not. Others have shown that overexpression of CDC20 indicates unfavorable prognosis and poor response to endocrine therapy in ER + breast cancer (Alfarsi et al., 2019; Tang et al., 2019); in contrast, we discovered that *CDC20* was related to worse prognosis only in the TNBC subtype, and we think that the different datasets that we analyzed result in the inconsistency. Other genes such as *BUB1*, *NUF2*, *CDC20*, *ASPM*, *KIF2C*, and *PRC1* have biological relevance to breast cancer progression, and *PLK1*, *NDC80*, and *CCNB2* only to TNBC progression, and these genes predict worse prognosis (Wang et al., 2015; Tang et al., 2019; Yang et al., 2019; Lv et al., 2020; Ren et al., 2020; Chen et al., 2021; Jiang et al., 2021; Koyuncu et al., 2021); likewise, we also found that these genes correlated negatively with prognosis in the TNBC subtype. Moreover, it is also reported that high expression levels of *AURKB*, *CDC6*, and *ECT2* suggest a poor prognosis for breast cancer (Mahadevappa et al., 2017; Daulat et al., 2019; Huang et al., 2019; Xiu et al., 2019), whereas we only found that CDC6 was negatively positively related to overall survival, and we consider that it is also the different datasets and analysis methods that cause the disparity. Furthermore, it is previously revealed that it might be an efficient therapeutic method to target *FOXM1* to impede advanced relapse and treat endocrine resistance (Roßwag et al., 2021). In addition, genes such as *SRSF1*, *BUB1B*, *KIF23*, and *HNRNPF* also play an important role in breast cancer, but so far, there is few reports on the association between these genes and the treatment or prognosis of breast cancer in the previous studies (Tyson-Capper and Gautrey, 2018; Du et al., 2021; Jian et al., 2021; Koyuncu et al., 2021); inspiringly, we performed it and obtained an exact result in our study. Also, we newly introduced applicant genes such as *RPA1* and *CDC5L* in TNBC, but their mechanism remains to be discovered with exploratory studies.

In terms of the pathway enrichment analysis, the nodes represent the pathway, while the edges mean that there is a functional similarity between the two pathways. As was consistent with the abovementioned analysis, the pathway enrichment analysis also showed that TNBC is the most complex subtype in breast cancer. In addition, there is an overlap of the pathways in the TNBC, Her2, and luminal B subtypes, while the pathways enriched in the luminal A subtype were unique. This suggested that the luminal A subtype occurs in a completely different way. If so, the treatment of the luminal A subtype should be different from the other three subtypes, especially the postsurgical adjuvant therapy, or in other words, there should be a specific treatment strategy to be formulated just for the luminal A subtype compared with the others. Of course, pathways about DNA replication and DNA repair are only included in TNBC, which means studies focused on these pathways can help shed light upon TNBC and develop treatments that are only indicated for TNBC. Also, for the same reason, patients of TNBC, Her2, and luminal B subtypes may benefit from studies on mitosis in the future. Otherwise, in addition to pathways related to DNA replication, DNA repair, and mitosis, the ERBB4 signal was only enriched in TNBC, while the NOTCH4 signal was only in the Her2 subtype, and chemokine and kinesins in both of them, compared with the luminal B subtype. ERBB4, a member of the human epidermal growth factor receptor family, has been previously reported to be a valuable prognostic marker when united with the pathologic stage in TNBC and may be helpful in predicting the therapeutic efficacy for TNBC (Kim et al., 2016). However, the biological function of ERBB4 and its potential as a cancer drug target have not been explicitly described, and we also hope that patients suffering from TNBC with ERBB4 overexpression would benefit from further clinical trials on receptor tyrosine kinase (RTKs). NOTCH4 has been identified to hinder differentiation, functional development, and branching morphogenesis of the mammary epithelium (Uyttendaele et al., 1998). In breast cancer, NOTCH4 is predominantly expressed in the Her2 subtype, and the expression is also discovered to be associated with bad prognostic factors (Wang et al., 2018). As the NOTCH4 signal pathway is found to be enriched in the Her2 subtype in our study, using NOTCH4 antagonists to suppress NOTCH4 signaling may be a novel and individually distinct strategy to treat Her2 subtype breast cancer.

According to the result of TF analysis, although most of the TFs have been reported to be associated with breast cancer, there were still TFs such as EOMES, POU3F2, NR3C1, and RUNX1 that were less reported in breast cancer. Studies focused on these TFs may shed light upon breast cancer in a new way and provide a novel therapeutic strategy. For microRNA analysis, the miR-875 family has been reported to serve as a marker for detection and prognosis in breast cancer (Liu et al., 2021). Others, such as miR-1284, miR-3613, and miR-208a families play a role in the progression of breast cancer (Zhang et al., 2019; Zou et al., 2019; Liu Y. et al., 2020). It is valuable to investigate other miRNAs in experimental studies.

The present research also includes some limitations. First, our study only looks at data in one dataset, and the GPL platform used in the dataset is now not universally applicable. Second, most of the clinicopathologic features are not included in the dataset, and we cannot rule out that these factors might have influenced our results. Third, we just predicted the TFs and miRNAs through DEGs and did not calculate the relationship between them or carry out experiments to validate it, but this is just what we are doing now.

## Conclusion

In this study, we preliminarily delved into the potentially comprehensive molecular mechanisms of breast cancer by creating a holistic view at the genomic and transcriptomic levels in different subtypes using computational tools. Our study introduced a network of genes, pathways, prognosis-related genes, TFs, and miRNAs which are possibly associated with a different subtype of breast cancer, and they can be good candidates for further analysis and provide novel approaches to treat breast cancer.

## Data Availability

The original contributions presented in the study are included in the article/Supplementary Material; further inquiries can be directed to the corresponding author.
