# Supplementary material for: A genomic and transcriptomic study toward breast cancer
Source: Front Genet. 2022 Oct 12;13:989565. doi: 10.3389/fgene.2022.989565 (PMC9596791; doi:10.3389/fgene.2022.989565)
Supplement: Supplementary file 1 [file Table1.DOC]

**Title: A genomic and transcriptomic study towards breast cancer**

**Raw data link:**  https://www.jianguoyun.com/p/DdNJ0oUQ_tPfChii_ckEIAA
